# Wildfire-driven thunderstorms cause a volcano-like stratospheric injection of smoke

**DOI:** 10.1038/s41612-018-0039-3

**Published:** 2018-08-20

**Authors:** David A. Peterson, James R. Campbell, Edward J. Hyer, Michael D. Fromm, George P. Kablick, Joshua H. Cossuth, Matthew T. DeLand

**Affiliations:** 1 Naval Research Laboratory, 7 Grace Hopper Avenue, Monterey, CA 93943, USA; 2 Naval Research Laboratory, 4555 Overlook Avenue SW, Washington, DC 20375, USA; 3 Science Systems and Applications, Inc. (SSAI), 10210 Greenbelt Road, Suite 600, Lanham, MD 20706, USA

## Abstract

Intense heating by wildfires can generate deep, smoke-infused thunderstorms, known as pyrocumulonimbus (pyroCb), which can release a large quantity of smoke particles above jet aircraft cruising altitudes. Injections of pyroCb smoke into the lower stratosphere have gained increasing attention over the past 15 years due to the rapid proliferation of satellite remote sensing tools. Impacts from volcanic eruptions and other troposphere-to-stratosphere exchange processes on stratospheric radiative and chemical equilibrium are well recognized and monitored. However, the role of pyroCb smoke in the climate system has yet to be acknowledged. Here, we show that the mass of smoke aerosol particles injected into the lower stratosphere from five near-simultaneous intense pyroCbs occurring in western North America on 12 August 2017 was comparable to that of a moderate volcanic eruption, and an order of magnitude larger than previous benchmarks for extreme pyroCb activity. The resulting stratospheric plume encircled the Northern Hemisphere over several months. By characterizing this event, we conclude that pyroCb activity, considered as either large singular events, or a full fire season inventory, significantly perturb the lower stratosphere in a manner comparable with infrequent volcanic intrusions.

## INTRODUCTION

Fire-triggered thunderstorms, or pyrocumulonimbus (pyroCb), are an extreme weather phenomenon associated with large wildfires at temperate latitudes. PyroCbs can release a large quantity of smoke particles into the lower stratosphere, often above the tropopause by several kilometers.^1,2^ The weather conditions driving pyroCb occurrence,^3^ along with increasingly active fire seasons,^4,5^ indicate that pyroCbs are a significant and endemic summertime feature in several regions worldwide. A single fire season in western North America, for instance, can include more than 25 intense single or multi-updraft pyroCb events.^6,7^ Since the discovery of pyroCb in the early 2000s, several stratospheric aerosol layers first thought to be of volcanic origin have been reclassified as originating from pyroCb activity.^2^ The significance of volcanic plumes in the lower stratosphere and their role in the climate system has been recognized for several decades. To date, however, the impact of pyroCbs on climate has never been systematically explored, and remains almost completely unquantified in recent studies of the lower stratosphere.^8–10^

Here, we quantify the mass of smoke aerosol particles injected into the lower stratosphere from five near-simultaneous intense pyroCbs observed in western North America on 12 August 2017, referred to hereafter as the “Pacific Northwest Event”. Systematic satellite observations are employed to examine the evolution of the event and to obtain essential input parameters, including the spatial extent and vertical profile of the pyroCb plume within the lower stratosphere. This study establishes an observational benchmark for extreme pyroCbs, with the goal of motivating modeling and in situ study of lower-stratospheric plume composition, chemical and physical particle evolution, and radiative properties that may influence lower-stratospheric chemistry and dynamic circulation on seasonal and hemispheric scales.

## RESULTS

### Stratospheric smoke mass estimates

**Fig. 1:**
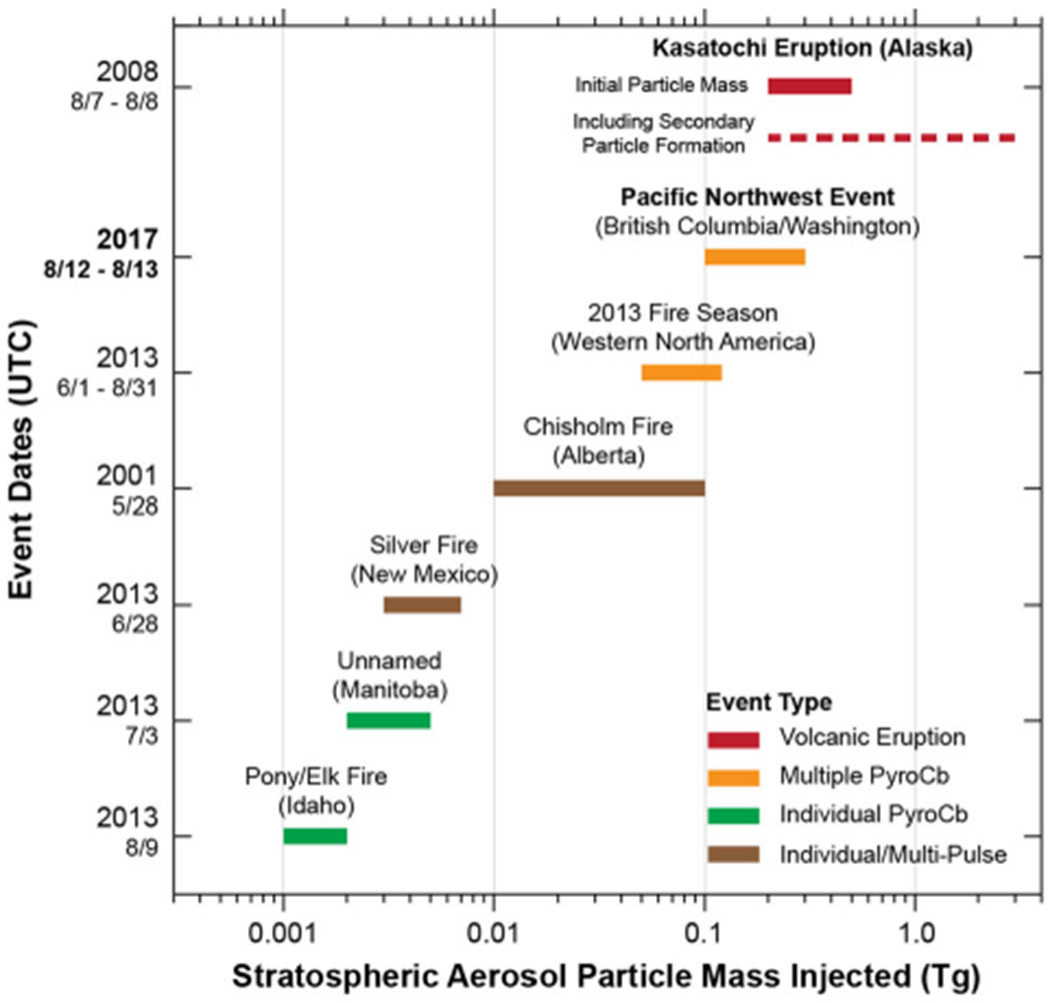
Comparisons with stratospheric particle mass estimates from other significant events. Bars indicate the approximate uncertainty range of stratospheric aerosol particle mass injected. All mass estimates are displayed using a logarithmic scale (*x*-axis). Color scheme indicates event type and characteristics

The stratospheric smoke intrusion from the Pacific Northwest Event was associated with five distinct pyroCb updrafts. Calculations based on the combination of lidar and passive remote sensing observations reveal that this event injected an estimated 0.1–0.3 Tg of total aerosol particle mass into the lower stratosphere (Fig. 1). The Pacific Northwest Event was comparable to the total stratospheric particle mass injected by the initial plume of a moderate volcanic eruption, characterized by a Volcanic Explosivity Index (VEI) between 3 and 4.^9,10^ The Kasatochi eruption (VEI of 4) in the Aleutian Islands of Alaska (United States, 7–8 August 2008) serves as a suitable reference event, given its proximity in latitude, occurrence during the same month, and injection to similar stratospheric altitudes. Kasatochi yielded an estimated 0.2–0.5 Tg of stratospheric aerosol particle mass during its initial eruptive cycles (see Methods for details). This plume was accompanied by approximately 1.2–2.2 Tg of sulfur dioxide (SO_2_) vapor mass.^11,12^ Total accumulated stratospheric particle mass caused by Kasatochi, accounting for the secondary conversion of SO_2_ to sulfate-based compounds over the weeks and months post-eruption,^13^ ranged between 2.0 and 3.0 Tg.

The stratospheric smoke injection from the Pacific Northwest Event was an order of magnitude larger (0.1 vs. 0.01 Tg) than the most significant single-event stratospheric intrusion of smoke aerosol recorded to date (Fig. 1), associated with multiple pyroCb updrafts (pulses) observed during the Chisholm Fire in Alberta, Canada in 2001.^1^ The Pacific Northwest Event also likely exceeded the total aerosol particle mass injected by all pyroCb activity (26 events) inventoried during the fire season of 2013 across western North America.^7^ Details of the extreme fire behavior and pyroCb activity observed during 2013 have been examined previously.^3,6^ Stratospheric intrusions from each individual pyroCb, such as those associated with the Silver and Pony/Elk fires, were two orders of magnitude lower in total smoke particle mass than the Pacific Northwest Event. However, the cumulative seasonal impact (0.04–0.12 Tg over 3 months) is still comparable to a relatively small volcanic eruption on its own.

### Evolution of the Pacific Northwest Event

**Fig. 2:**
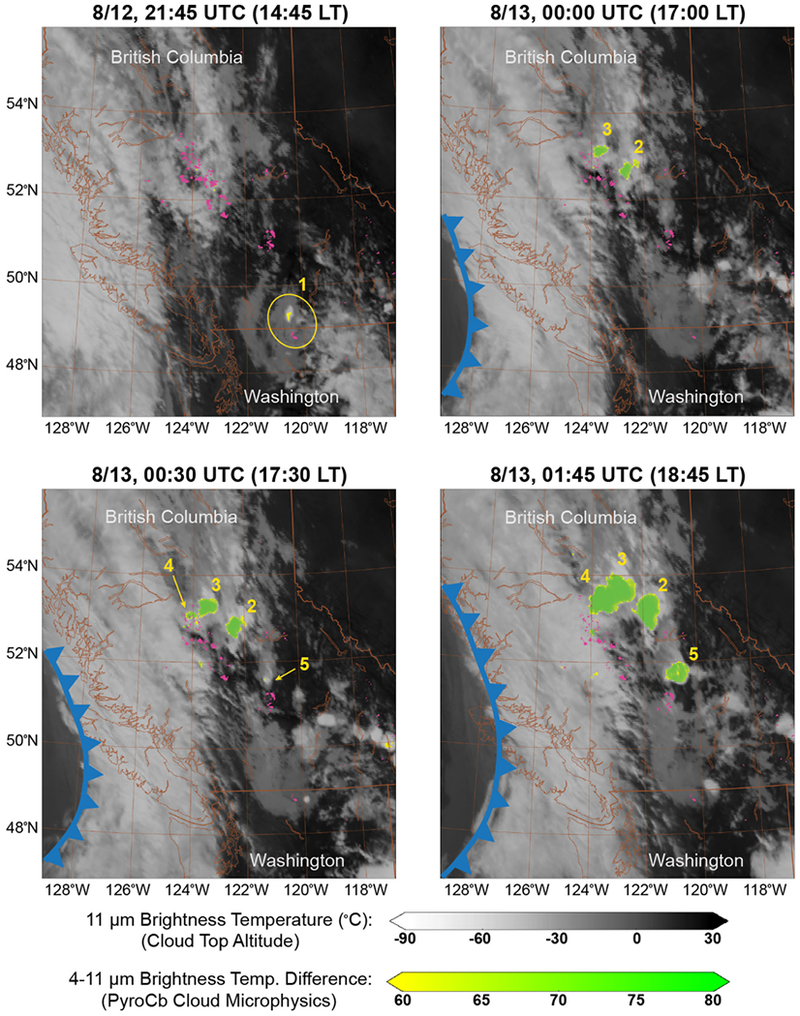
Evolution of the 12 August 2017 Pacific Northwest pyroCb event from satellite. Grayscale shading indicates the thermal infrared (11 μm) brightness temperature (GOES-16), with colder, high-altitude cloud tops displayed in white. Green shading indicates the solar reflectivity of smaller cloud top particles relative to the 11 μm brightness temperature. PyroCb smoke particle perturbations therefore correspond with larger green values. Pink shading indicates all satellite fire detections for the preceding 24 h in native resolution (MODIS). The approximate position of the approaching surface cold front is highlighted in blue

Each pyroCb contributing to the 12 August Pacific Northwest Event was detected by the constellation of Geostationary Operational Environmental Satellites (GOES) observing North America^7^ (Fig. 2). The first pyroCb occurred over the Diamond Creek Fire (https://inciweb.nwcg.gov/incident/5409/) in northern Washington State, United States at 21:00 UTC (14:00 local time [LT]). The four remaining pyroCb were caused by a broad complex of wildfires in British Columbia, Canada, with the second and third pyroCb developing at 23:00 UTC (16:00 LT), the fourth at 00:00 UTC (17:00 LT), and the fifth at 00:30 UTC (17:30 LT). All five pyroCb updrafts were active for 1–4 h over a total 5 h period, which is comparable to the duration of the Kasatochi eruption.^11^ Estimated stratospheric mass (0.1–0.3 Tg) is higher than the range of estimates for total dry particulate mass derived from the area burned by these fires (0.02–0.26 Tg). However, this latter estimate does not account for the total amount of condensed water mass simultaneously lofted into the stratosphere by each updraft or secondary particle formation within the plume.

During the preceding days, an intensifying upper-level cyclone and surface cold front approached the west coast of North America. Wildfire behavior in the Pacific Northwest intensified ahead of this disturbance, driven by enhanced southwesterly surface winds and a relatively dry surface air mass, peaking on the afternoon of 12 August (Fig. 2). The approaching storm also induced transport of relatively moist and unstable air from the nearby Pacific Ocean over the dry, deep, and unstable nearsurface boundary layer, thus providing a mechanism for large-scale rising motion in the middle and upper troposphere. These combined characteristics are a potent recipe for high-based convection (relatively dry thunderstorms over elevated terrain), which is consistent with a conceptual model for pyroCb development.^3^ Strong thermal buoyancy induced by the large and intense wildfires sustained smoke column updrafts necessary to penetrate the tropopause, thus providing a direct pathway for smoke aerosols to enter the lower stratosphere. At least two smaller pyroCbs occurred in the same area on 11 August, but they were not as deep as the 12 August Pacific Northwest Event, reinforcing the importance of meteorological contex in supporting deep pyroCb updrafts.

PyroCb cloud tops are characterized by relatively small particles induced by significant smoke aerosol loading and the concomitant seeding/nucleation from ambient water vapor.^14,15^ The relative reduction in cloud particle size compared with more typical regional thunderstorms causes an unusually large solar reflection by pyroCb cloud tops in the GOES shortwave infrared channel (4 μm). This characteristic is used to distinguish the pyroCb clouds in Fig. 2 through subtraction of the 11 μm thermal infrared brightness temperature from that at 4 μm. This brightness temperature difference (BTD) highlights the pyroCb clouds, and reveals that the four Canadian pyroCbs dominated the event. The Canadian pyroCbs also exhibited the lowest observed 11 μm cloud top brightness temperatures (below −60 °C, not evident in Fig. 2), indicating relatively stronger convective updrafts. Relating these cloud-top brightness temperatures to height using local thermodynamic profiles from radiosondes (weather balloons) reveals that these cold cloud tops reached altitudes of 11.5–12.5 km, which exceeded the local cold-point tropopause. Each of the four Canadian pyroCbs contributed an estimated 0.03–0.08 Tg to the initial total particle mass (0.1–0.3 Tg), which is roughly comparable to experiencing four Chisholm pyroCb events on a single afternoon (Fig. 1).

### Characteristics of the stratospheric smoke plume

**Fig. 3:**
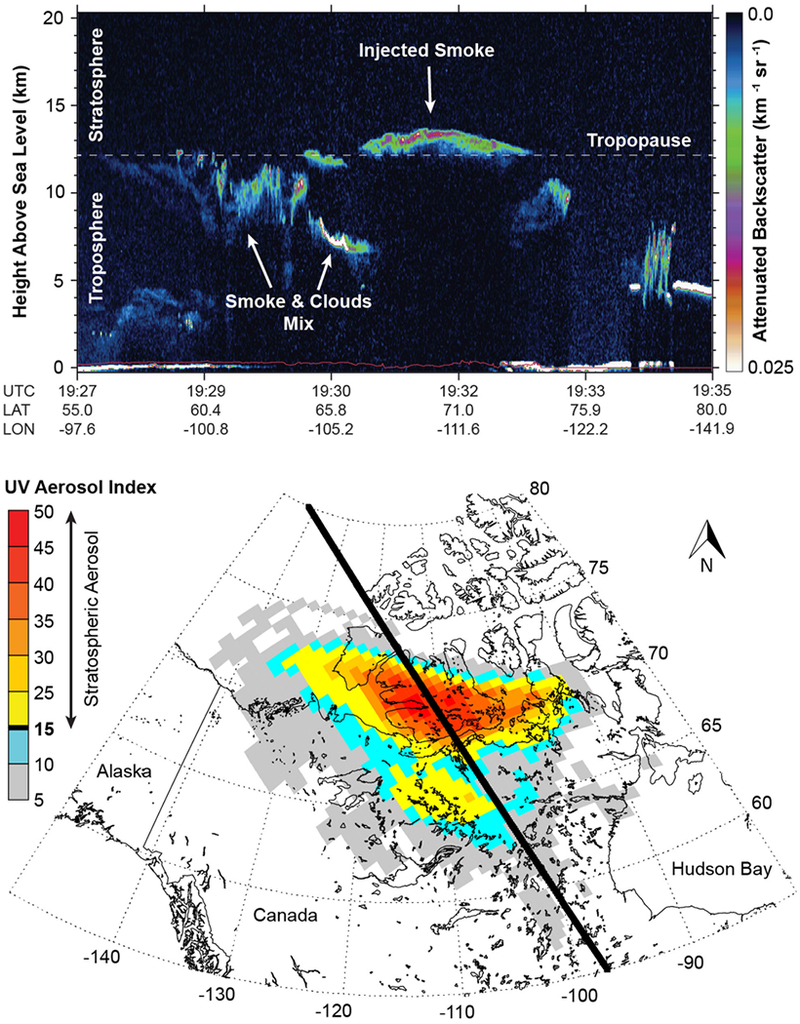
Characteristics of the young stratospheric smoke plume. Top panel shows profiles of 532 nm attenuated backscatter (km^−1^ sr^−1^) observed by CALIOP on 14 August 2017 for a daytime (ascending) CALIPSO overpass beginning 19:27 UTC. The dashed white line denotes the approximate tropopause altitude. Bottom panel shows near-coincident ultra-violet (UV) aerosol index (AI) observations from OMPS, with the CALIPSO satellite track superimposed in black. The horizontal extent of the stratospheric smoke plume (AI ≥ 15) is displayed in shades of yellow and red

An expansive thunderstorm outflow anvil cloud, comprised of both smoke and water ice, persisted for nearly 12 h in GOES infrared imagery after cessation of the parent pyroCb updrafts. The Cloud-Aerosol Lidar with Orthogonal Polarization (CALIOP), flown aboard NASA’s polar-orbiting CALIPSO satellite,^16^ passed over the decaying cloud shield approximately 8 h after pyroCb cessation (10:45 UTC on 13 August), confirming the injection of smoke and water ice at least 1 km into the stratosphere (not shown). By the afternoon of 14 August (19:30 UTC), ice crystal influence on the CALIOP backscatter profile within the plume had diminished, and a distinct 1.5 km deep residual layer was present over northern Canada (Fig. 3). CALIOP attenuated laser backscatter measurements were converted to smoke particle mass density averages over the two successive orbital passes incident upon the young stratospheric plume at this time, providing an estimated range of 73–220 μg m^−3^ (see Methods).

The horizontal extent of the stratospheric plume was estimated by applying the ultra-violet Aerosol Index (AI, dimensionless) retrieved from Ozone Mapping Profiler Suite (OMPS) Nadir Mapper (NM), flown aboard the Suomi National Polar-orbiting Partnership (S-NPP) satellite.^17^ AI is sensitive to the altitude of the light-absorbing smoke particles that make up pyroCb plumes (e.g., black and brown carbon), with stratospheric layers corresponding to the largest values (e.g., >15–20).^1^ On 14 August, AI values measured over a large portion of northern Canada exceeded those of any known pyroCb plume on record^2^ (Fig. 3). Comparison with coincident CALIOP profiling showed that pixels with an AI at or above 15 were consistent with smoke particles in the stratosphere, which mirrors the AI threshold applied to the 2001 Chisholm pyroCb smoke plume^1^ (Fig. 1). Integration of each individual pixel area coinciding with stratospheric smoke particles yielded an instantaneous plume area of nearly 800,000 km^2^.

### Stratospheric smoke transport and residence time

**Fig. 4:**
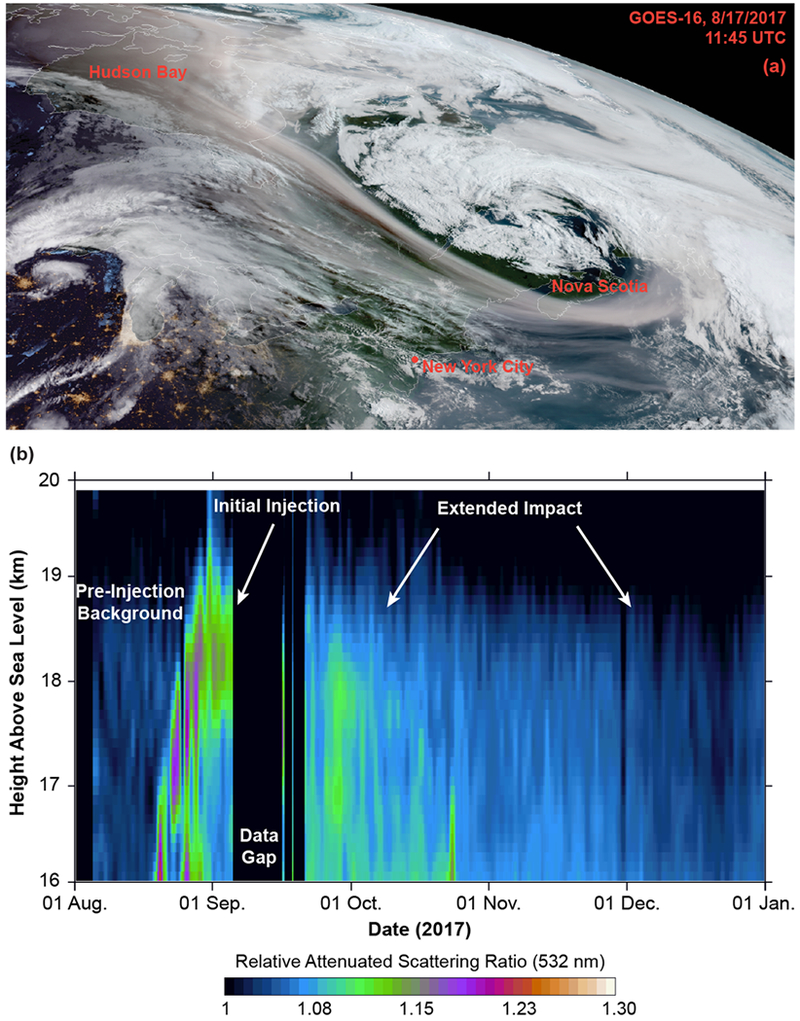
Stratospheric smoke transport and residence time. Top panel shows true color imagery from the GOES-16 GeoColor Algorithm (developed by CIRA) near sunrise on 17 August 2017 (11:45 UTC). The stratospheric smoke plume extends from Hudson Bay to the northern Atlantic Ocean. Bottom panel provides a time series of CALIOP relative attenuated 532 nm scattering ratio averaged over the Northern Hemisphere between 40°N and 80°N. The gaps in the time series represent CALIOP data outages in early August and mid-September 2017

The plume was transported downwind of North America by relatively strong winds associated with the upper-tropospheric jet stream, which also influences the circulation of the lower stratosphere. Figure 4a shows a true color satellite image (GeoColor) from the Advanced Baseline Imager (ABI) onboard the GOES-16 satellite over eastern North America on 17 August (image used with permission from the Cooperative Institute for Research in the Atmosphere [CIRA]). Smoke from the Pacific Northwest Event is evident between two mid-latitude cyclones aligned with the upper-tropospheric jet stream, extending from the Canadian Arctic to the Atlantic Ocean, near the coast of Maine and Nova Scotia. This high-altitude plume reached Europe by 19 August, Asia by 24 August, and encircled the entire Northern Hemisphere by 31 August (not shown).

A time-height analysis of CALIOP relative attenuated molecular scattering ratio averaged over the Northern Hemisphere between 40°N and 80°N shows background values near 1.0 in the lower stratosphere (16–20 km) prior to the Pacific Northwest Event (Fig. 4b). The initial impact of the Pacific Northwest Event is evidenced by its rapid increase above 1.2 from mid-August to early September. Scattering ratios remained higher than background through the middle of December, suggesting that significant levels of smoke remained in the lower stratosphere for approximately 4 months.

In the absence of any significant volcanic particle mass reaching the stratosphere between August and December 2017, as well as any additional extreme pyroCb events, the prolonged period of elevated scattering ratios highlighted in Fig. 4b reflects a sustained perturbation of the lower stratospheric aerosol layer from the 12 August Pacific Northwest Event. Similar characteristics were observed following the Kasatochi eruption.^10^ The Pacific Northwest Event therefore constitutes a stratospheric intrusion similar to a moderate volcano, including total aerosol particle mass initially injected, the nature of its downwind transport, and seasonal persistence.

## DISCUSSION

This study provides a unique quantitative analysis of the aerosol particle mass injected into the lower stratosphere by a single extreme pyroCb event, revealing that its impact is comparable in magnitude to the initial stages of a moderate volcanic eruption. The cumulative stratospheric impact from all pyroCb activity observed during the fire season of 2013 in western North America was also significant. These results indicate that pyroCb activity, occurring as either large singular events or smaller events accumulated over a fire season, influence the lower stratosphere in a manner consistent with infrequent volcanic intrusions. Meteorological conditions driving pyroCb development suggest that stratospheric intrusions of smoke particles can be expected every fire season in favored regions of the Northern Hemisphere.^3^ The expansive stratospheric plume associated with the Australian “Black Saturday” pyroCb event^18^ (7 February 2009) shows that pyroCbs can also impact the Southern Hemisphere.^19^ While the community already monitors surface-based and lower tropospheric aerosol and chemical perturbing agents that influence the lower stratosphere, such as volcanic eruptions and halogen compounds, the Pacific Northwest Event demonstrates that pyroCbs also likely play a significant role in the climate system.

The physical particle properties and chemical evolution of pyroCb smoke in the stratosphere remain highly uncertain, as do their optical characteristics and the potential for significant solar dimming effects. PyroCb smoke is comprised primarily of carbonaceous aerosol, with physical and optical properties very different from volcanic plumes comprised of ash and sulfate-based particles. Processing of pyroCb smoke during the lofting process into the stratosphere will change its composition and properties relative to surface or tropospheric smoke plumes. In addition, since pyroCb updrafts begin with strong surface inflow winds in a dry environment, additional aerosol particles such as mineral dust, may also be contributing factors.^20,21^ Detailed airborne sampling, in combination with ground and spaceborne observations, is therefore essential for improved understanding of pyroCb impacts on chemistry, radiation, secondary particle formation, and dynamic circulation. This research is further motivated by the recent increase in large wildfires observed across many boreal and temperate ecosystems,^4,5^ which suggests that stratospheric intrusions of smoke aerosol from pyroCbs may also be increasing.

## METHODS

### PyroCb detection

PyroCb detection for the 2017 Pacific Northwest Event was based on the Advanced Baseline Imager (ABI) onboard the recently launched GOES-16 satellite. The ABI provides higher spatial resolution and temporal sampling compared with the previous generation of GOES sensors. Close proximity to one or more satellite-based fire detections (within 40–60 km) is the first requirement for pyroCb detection.^7^ This study is based on the fire products for the MODerate Resolution Imaging Spectroradiometer (MODIS) sensors aboard the Terra and Aqua satellites (MYD14/MOD14, collection 6).^22^ PyroCb detection was initiated only for areas with MODIS fires detected during a 24-h period preceding each GOES-16 observation.

To be classified as pyroCb, a cloudy pixel must exhibit a thermal infrared (11 μm) brightness temperature below an approximated homogeneous liquid-water freezing threshold,^14^ which implies a high likelihood of vertical cloud development near the tropopause altitude. Extreme smoke aerosol loading and strong updrafts within a pyroCb induce a discernable microphysical shift (from indirect aerosol effects) toward smaller cloud droplets and ice particles when compared with those of typical convective storms occurring over elevated terrain.^15^ This effect is resolved from satellite by observing a relatively large daytime near-infrared (4 μm) reflectivity.^14^ Application of a 4 and 11 μm BTD therefore allows for distinction of pyroCb clouds from traditional convection.^7^ This methodology relies on reflected sunlight, but captures the overwhelming majority of pyroCb activity, since initiation after sunset is relatively infrequent.^2,3^ Testing with previous GOES imagers (e.g., GOES-15) has demonstrated the successful detection of individual pyroCb events, pyroCbs embedded within traditional convection, and multiple, short-lived pulses of activity.^7^ Imagery products based on these methods are posted in near-real-time on the Naval Research Laboratory’s pyroCb website: http://www.nrlmry.navy.mil/pyrocb-bin/pyrocb.cgi.

### Stratospheric smoke mass

Quantifying the total particle mass of the stratospheric aerosol layers injected by pyroCbs involves the combination of active and passive remote sensing techniques. Each calculation was based on observations 24–48 h after pyroCb cessation, when the stratospheric plume was comprised primarily of residual smoke aerosol rather than ice particles. Visible and infrared imagery from the GOES-16 ABI was examined to confirm that the study region was devoid of cloud contamination. Regional tropopause heights were determined using temperature profiles from local radiosondes. The vertical extent of the plume above the tropopause was constrained using vertical profiles of 532 nm backscatter and linear laser depolarization ratio from the CALIOP.^16^ The average particle mass density (*M_ρ_*) of the stratospheric smoke layer was calculated by

$$M_{\rho }=\frac{\beta R}{\varepsilon },$$

where *β* is the average CALIOP Level 1 backscatter (units of m^−1^ sr^−1^), *R* is an assumed particulate extinction-to-backscatter lidar ratio (units of sr), and *ε* is the particle mass extinction coefficient (units of m^2^ g^−1^).

The calculation of *M_p_* is sensitive to the values of *ε* and *R*, which are dependent on the physical and optical properties of smoke particles. While smoke plumes from temperate and boreal forest fires have been extensively studied,^23,24^ particles lofted by pyroCbs are transported to the stratosphere through an environment of ice phase or possibly mixed-phase condensation. This process will likely alter the effective particle properties significantly from those observed at ground level. The amount of mineral dust lofted within pyroCb plumes and its impact on optical properties also remains unresolved.^20,21^ An estimated range of 3.0–6.0 m^2^ g^−1^ was therefore used for *ε*, based on the available literature for aged boreal smoke plumes^25^ and accounting for potential entrainment of mineral dust.^26^ Similarly, a range of 40–60 sr was used for *R* to account for a potential mix of smoke particles, water/ice, and mineral dust.^27^

The horizontal area of the pyroCb plume was constrained using the ultra-violet (UV) AI (dimensionless) retrieved from the Ozone Mapping Profiler Suite (OMPS) Nadir Mapper (NM), which is sensitive to the altitude of light-absorbing aerosols.^1^ All OMPS pixels along each CALIOP overpass track were examined to derive an AI threshold for stratospheric aerosol presence (AI ≥ 15), which mirrored the value applied to the 2001 Chisholm pyroCb smoke plume.^1^ By assuming a constant altitude of the stratospheric smoke plume, this AI threshold was applied to identify all pixels that contain stratospheric smoke particles. Integration of each individual pixel area (OMPS pixels are ~50 km × 50 km at nadir) provided the total area of the stratospheric smoke plume. Integration of *M_ρ_* over the smoke plume depth and area provided an estimate of the total mass of smoke aerosol injected into the stratosphere by the pyroCb event.

Adjustments were required to account for the fully attenuating smoke particle layer of the Pacific Northwest Event. An average smoke plume layer depth of 1.5 km was employed and *M_ρ_* was augmented by ~10% (based on increasing *β*) to account for additional smoke particles between the detection limit and the tropopause altitude. CALIOP backscatter data at 1064 nm (not shown) support this augmentation.

As a closure experiment, radiative transfer calculations were employed to produce simulated AI values from the inputs for the CALIOP-based smoke particle mass estimates, including the range for *ε* and observed plume depth. AI was simulated using the OMPS method for two UV radiances (330 and 390 nm) computed by the Santa Barbara DISORT Atmospheric Radiative Transfer (SBDART) model.^28^ Particulate single scatter albedo and asymmetry parameters were assumed to be 0.9 and 0.6, respectively, with an Angstrom exponent of 1.6.^29^ This simulation produced AI values of 15–20, which match the pyroCb AI threshold for stratospheric smoke particles, thus reinforcing confidence in the CALIOP-based aerosol particle mass estimates. The simulated AI varies between −10 and +15% when choosing different optical properties relevant for a smoke and ice plume mixture in the UV.

Calculations of the total smoke particle mass emitted by the fires contributing to pyroCb development were based on burned area observations derived from satellite and airborne observations. The Canadian fires burned 300,000 hectares during 9–16 August 2017 (http://cwfis.cfs.nrcan.gc.ca/report/archives?year=2017&month=08&day=16&process=Submit). The Diamond Creek fire in Washington State burned over 10,000 hectares (https://inciweb.nwcg.gov/incident/5409/). Total area burned on 12 August 2017 was estimated at 25–75% of each total, based on the tendency of extreme fire days to dominate total area burned in boreal regions.^30^ Fuel consumption was estimated in a range of 22–53.2 Mg per hectare, and smoke emissions (PM_2.5_) per fuel consumed were estimated in a range of 9.4–21.2 g kg^−1^. ^31^ Combining these three factors and their associated ranges provided a range of the total dry smoke particle mass released on 12 August (0.02–0.26 Tg).

Weather and fuel conditions consistent with the rapid fire growth observed for these fires are consistent with high fuel consumption and smoke production per area burned, but this effect is poorly quantified. Surface-based estimates were therefore calculated using the full climatological range of fuel consumption and smoke release for fires in the study region. While these emissions factors include some secondary particle production in the plume,^32^ they exclude coarse particles and sulfate aerosol particles converted from gaseous SO_2_. Literature estimates indicate these two components together contribute less than 15% to total particulate mass.^24,31^

### 2013 fire season

An inventory of pyroCb activity was developed for western North America using the fire season (June–August) of 2013.^7^ It contains 26 individual events, comprised of 31 individual pulses of intense pyroCb activity (e.g., separate updrafts/anvil clouds), all of which were capable of injecting smoke particles into the upper troposphere and/or lower stratosphere. A first-order estimate of the stratospheric smoke particle mass injected across the entire inventory was derived based on three pyroCb events that spanned the spectrum of observed activity during 2013. For example, the Pony/Elk Fire in Idaho (United States) on 9 August was a relatively small pyroCb. A wildfire complex in Manitoba, Canada produced a larger pyroCb on 3 July. The Silver Fire in New Mexico (United States) produced three pulses (convective updrafts) of pyroCb activity in rapid succession, and was likely the most extreme event in the sample. Each of these three events produced a stratospheric plume of aerosol particles that was well observed by CALIOP and OMPS from 24 to 48 h after initial injection. Stratospheric particle mass estimates were produced for each case using the same methodology as the 2017 Pacific Northwest Event. The range of mean mass estimates for these three events (based on *ε* and *R*) was used as an estimate for the remaining pyroCb events in the inventory. Summation over the full inventory provided the total seasonal stratospheric smoke particle mass injected.

### Volcanic aerosol mass

Similar to the pyroCb methodology, stratospheric aerosol mass particle estimates for the eruption of Mt. Kasatochi (Alaska, 7–8 August 2008) began with CALIOP observations nearly 48 h after the eruption (at least one complete diurnal cycle). Tropopause heights were determined using temperature profiles from the closest radiosonde observations to the plume. However, several modifications were required. Mass estimates for the initial eruptive plume injection account for two layers of stratospheric aerosol particles observed at 11.5–13 and 15–16 km.^11^ A range of 0.6–0.8 m^2^ g^−1^ was used for *ε*, based on observations of volcanic plumes within 1–3 days of an eruption.^33^ The value for *R* was fixed at 60 sr.^34^ Initial estimates based on these methods (0.4–0.5 Tg) are consistent with particulate mass estimates retrieved independently from infrared satellite sensors within 48 h of the Kasatochi eruption.^35^

The Kasatochi stratospheric plume of aerosol particles was accompanied by approximately 1.2–2.2 Tg of sulfur dioxide (SO_2_) vapor mass.^11,12^ Conversion of this SO_2_ component (molar mass = 64) to sulfates in the lower stratosphere increases the total particulate mass estimate over the ensuing weeks and months post-eruption.^36^ To estimate this additional mass, it was assumed that 75% of the SO_2_ converted to sulfuric acid (molar mass = 98) and 25% to water,^13^ producing a total accumulated stratospheric particle mass estimate between 2.0 and 3.0 Tg.

The lower bound of the initial particle mass estimated from CALIOP (0.4 Tg) was also modified to account for potential SO_2_ to sulfate-based secondary particle formation between the initial injection and the CALIOP observation. Over a single diurnal cycle, we estimate a conversion of 0.1 Tg of SO_2_ vapor to 0.2 Tg particle mass.^12,13^ When subtracted, this yields a 0.2 Tg lower bound value for the initial stratospheric injection of Kasatochi. Neither the lower nor upper-bound estimates consider potential particle fallout during this period.

### CALIOP data processing

Stratospheric relative 532 nm attenuated molecular scattering ratios were derived from CALIOP based on methods described in the available literature.^37^ Level 1B attenuated 532 nm lidar backscatter (km^−1^ sr^−1^) data were aggregated at 1-day intervals for all measurements collected within the lower stratosphere (16–20 km above mean sea level) over the Northern Hemisphere between 40°N and 80°N. Molecular profiles were derived using Goddard Modeling and Assimilation Office (GMAO) Goddard Earth Observing System Model, Version 5 (GEOS-5), meteorological reanalysis thermal profiles included in the CALIOP Level 1 files. The relative attenuated molecular scattering ratio is defined as total attenuated particle backscatter normalized by the corresponding attenuated molecular profile. Ozone is not explicitly defined, however, nor is any coincident aerosol transmission occurring between 20 and 35 km, and thus these profiles are specifically defined as relative. To suppress noise, these data were passed through a Gaussian smoothing filter, the so-called *“Von Hann Window”*, and rendered at 25 m vertical resolution and 0.05-day temporal resolution using corresponding spatial and temporal half-widths of 375 m and 1.0 days.

### Data Availability

The CALIOP data that support the findings of this study are available from https://eosweb.larc.nasa.gov/project/calipso/calipso_table. OMPS data are available from https://ozoneaq.gsfc.nasa.gov/data/omps/. GOES satellite data are available from NOAA CLASS archive https://class.noaa.gov. Imagery products based on GOES data, including the pyroCb detection product, are posted in near-real-time on the Naval Research Laboratory’s pyroCb website: http://www.nrlmry.navy.mil/pyrocb-bin/pyrocb.cgi. The standard MODIS fire products (MOD14) can be obtained from https://lpdaac.usgs.gov/dataset_discovery/modis/modis_products_table/mod14_v006. Derived stratospheric mass estimates are available from the corresponding author upon request.
